# Supplementary material for: Learning curve and surgical outcome of robotic assisted colorectal surgery with ERAS program
Source: Sci Rep. 2022 Nov 29;12:20566. doi: 10.1038/s41598-022-24665-w (PMC9709162; doi:10.1038/s41598-022-24665-w)
Supplement: Supplementary file 1 — Supplementary Information 1. [file 41598_2022_24665_MOESM1_ESM.zip › LSC Raw data-Ñ[▒K/116/O_ñw╝╨░O▒Kñσ.pdf]

正式摘要(電子簽章：000000209928570)

W65 / W65

臺中榮民總醫院

# Operation Note

[redacted]

- 手術日期：2021/02/05
- 手術醫師：VS 蔣鋒帆，CR 陳映菁，R 林志安，CR 黃俋霖
- Anesthesia : GE
- Position : Lithotomy
- Aseptic agents : Soap , Betadine Alcohol , Betadine Aqua

## Diagnosis

- Pre-operative diagnosis : Rectal cancer with multiple liver and lung metastasis and partial obstruction, cT4N1M1b
- Post-operative diagnosis : Ditto. s/p laparoscopic low anterior resection

## Surgical Indication

- 1.tumor resection and pathologic staging
  2.resolve partial obstruction

## Operation

- 1. Laparoscopic low anterior resection, end-to-end anastomosis, Endo-GIA 60-4.1mm*2, CDH 33mm.
  Bilateral doughnut: intact, air tight test: done, no air leak.
  2. High ligation (+) above the left colic artery.
  3.r/o peritoneal seeding, s/p resection and sent to pathologic prove
  4. Cut margin: proximal 10 cm, distal 2 cm.
  5. Take down of splenic flexure: (-).
  6. A 12mm penrose drain placed in the pelvis.

## Finding

- 1. A 3.5*3 cm Broman type II tumor at lower rectum, which invaded to the serosa. With enlarged lymph node, cT4N1M1.
  2. There were liver metastases or possible peritoneal seeding.
  3. There were no synchronous polyps or tumor at resected specimen.

## Procedure

- General endotracheal anesthesia was induced and then the patient was placed in modified Trendelenburg lithotomy position. Preoperative antibiotics were given. A Foley catheter and nasogastric tube were placed. The abdomen was prepped and draped in standard sterile fashion. A small vertical incision was made at midline and 12mm trochars were inserted into the peritoneal cavity by open method. Peumoperitoneum was established with CO2 inflation. Laparoscopy was inserted and then another three 5mm trochars were inserted over LUQ, LLQ and RUQ of abdomen. One 12mm trochar were inserted over RLQ. The operative findings were mentioned as above. The sigmoid colon was held by grasper and the peritoneum overlying the mesentery was scored with electrocautery. The inferior mesentery artery was identified, ligated above the left colic artery with endoclips, and transected. Dissection was done above the Gerota's fascia and toward the peritoneal attachments. The colon was mobilized from retroperitoneum overlying the sacral promontory and into the pelvis. The incision was extended laterally and medially across the peritoneal reflection. The mesorectum was then mobilized. Distal descending colon and rectum were mobilized. The rectum was divided with Endo GIA 60-4/1mm*1 distal tumor. The paraumbilical wound was enlarged and the rectum was pulled out. The distal descending colon was transected and the specimen was removed from the operative field. The cap of CDH was applied at the distal end of the descending

- colon. Pneumoperitoneum was constructed again. End-to-end anastomosis was established with CDH 33mm. Peritoneal tumor was resected and sent for pathologic proof. A 12 mm penrose drain was placed in pelvis. The fascia was closed with interrupted sutures of No.1 Vicryl. The subcutaneous layer was closed with 3-0 vicryl and 4-0 eutek. Skin was approximated with tissue glue. The patient tolerated the procedure well and was brought to the POR in stable condition.
- Estimated blood loss: minimum
- 器械數量: 正確
- 紗布數量: 正確

---

## Image

- PAINT
  - colon & rectum

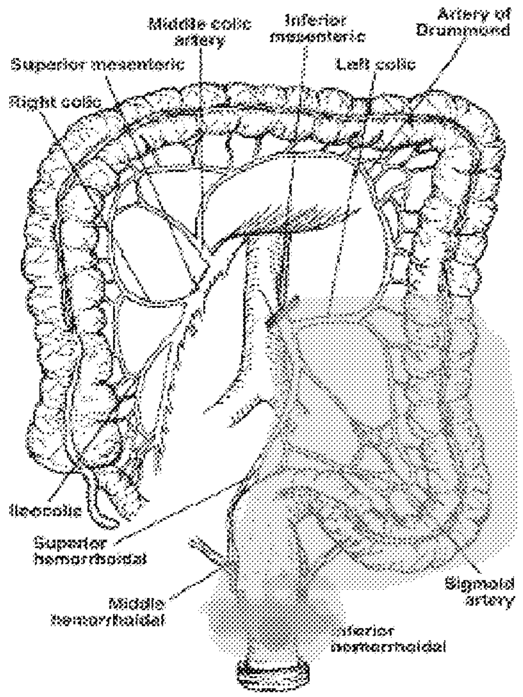


  - abdomen

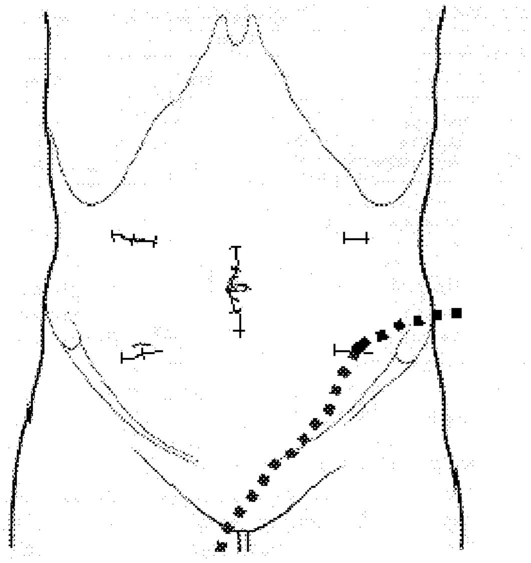

主任 2062F蔣鋒帆 / 醫師 2303K陳映菁

正式摘要(電子簽章：**000000209928570)**

| | |
|---|---|
| W65 / W65 | |

doctorRank:B
